# SNP-SNP interactions as risk factors for aggressive prostate cancer

**DOI:** 10.12688/f1000research.11027.1

**Published:** 2017-05-03

**Authors:** Venkatesh Vaidyanathan, Vijay Naidu, Nishi Karunasinghe, Anower Jabed, Radha Pallati, Gareth Marlow, Lynnette R. Ferguson

**Affiliations:** 1 Discipline of Nutrition and Dietetics, FM & HS, University of Auckland, Auckland, New Zealand; 2 Auckland Cancer Society Research Centre, Auckland, New Zealand; 3 School of Engineering,Computer and Mathematical Sciences, Auckland University of Technology, Auckland, New Zealand; 4 Department of Molecular Medicine and Pathology, FM & HS, University of Auckland, Auckland, New Zealand; 5 Experimental Cancer Medicine Centre, Cardiff University, Cardiff, UK

**Keywords:** prostate cancer, SNP genotyping, SNP-SNP interaction, SEQUENOM MassArray technology

## Abstract

Prostate cancer (PCa) is one of the most significant male health concerns worldwide. Single nucleotide polymorphisms (SNPs) are becoming increasingly strong candidate biomarkers for identifying susceptibility to PCa. We identified a number of SNPs reported in genome-wide association analyses (GWAS) as risk factors for aggressive PCa in various European populations, and then defined SNP-SNP interactions, using PLINK software, with nucleic acid samples from a New Zealand cohort. We used this approach to find a gene x environment marker for aggressive PCa, as although statistically gene x environment interactions can be adjusted for, it is highly impossible in practicality, and thus must be incorporated in the search for a reliable biomarker for PCa. We found two intronic SNPs statistically significantly interacting with each other as a risk for aggressive prostate cancer on being compared to healthy controls in a New Zealand population.

## Introduction

Prostate cancer (PCa) is highly prevalent, and around 1 in 6 patients are at risk of developing the aggressive form of the disease ^1^. It has become one of the most significant male health concerns worldwide ^2^. An individual is diagnosed as having high-risk or aggressive PCa based on the classification by the American Urological Association ^3^, when the clinical T stage ≥cT2c, and/or the Gleason score ≥8, and/or the serum prostate serum antigen (PSA) level >20ng/ml ^4^.

Although a hereditary aspect is well known for this disease ^5^, various studies have also shown that genetic interactions with biological and behavioral factors play an important role in the overall risk and prognosis of PCa ^6– 8^. Variations in the genome are a major contributor to the differences in disease susceptibly amongst individuals ^9^. Single nucleotide polymorphisms (SNPs) are the most commonly identified variations in a genome.

Analysing the role of SNP-SNP interactions and epistasis ^10^ is very appealing among researchers working on risk factors for various cancers ^11– 13^, including prostate cancer ^14^. Here we have identified a SNP-SNP interaction as a risk factor for aggressive PCa, by comparing the data generated after carrying out SNP genotyping using the SEQUENOM MassARRAY iPLEX ^®^ assay, and the TaqMan ^®^ assay (depending on the gene of interest) from the DNA extracted from blood samples. These samples were taken from a New Zealand cohort of men with self-reported European ethnicity that have been clinically diagnosed with aggressive and non-aggressive PCa, and healthy controls with no reported symptoms of the disease. Symptoms include increased urination during night time along with a frequent urge to urinate problems maintaining a steady flow of urine, hematuria and dysuria ^15^. Our results indicate a strong influence of gene x environment interaction in overall gene expression and epistasis.

## Methods

### Study population

Patients with a clinically established diagnosis of PCa (aggressive and non-aggressive) from the Auckland Regional Urology Registry (Auckland, Middlemore, and North Shore hospitals), and certain private practices in the Waikato region of New Zealand were sent invitations along with the written consent forms to participate in this study between the years 2006 and 2014. Eventually, a total of 254 patients with various grades of PCa voluntarily participated in our study after providing us with written informed consent. (Ethics reference NTY05/06/037 by Northern B Ethics Committee, New Zealand, previously Northern Y Ethics Committee, New Zealand). Additionally, 369 males from the Auckland region of New Zealand with no reported symptoms of PCa were considered as healthy controls for this study (Ethics reference NTY/06/07/AM04 by Northern B Ethics Committee, New Zealand, previously Northern Y Ethics Committee, New Zealand), recruited by advertising in and around the University of Auckland. Written informed consent for participation in the study was also obtained from the male healthy controls.

Each individual participating in this study completed a demographic and lifestyle questionnaire. Because of the influence of age in this disease ^16^, care was taken to invite men to participate in this study that were between the ages of 40 to 90 years at the time of diagnosis for patients with PCa, and at the time of recruitment for healthy controls. ( Dataset 1 ^17^). The average age of men with aggressive PCa was calculated to be 66 years, 67 years for men with non-aggressive PCa and 58 years for healthy controls.

### Collection and processing of blood samples

Patient blood samples were collected at respective outpatient clinics at the Auckland, North Shore and Counties Manukau Hospitals, New Zealand. The blood samples of healthy controls were collected at the Faculty of Medical and Health Sciences, the University of Auckland, New Zealand and the New Zealand Blood Bank, Great South Road Centre, Epsom, Auckland New Zealand.

Blood samples from each participant were collected in Vacutainer ^®^ tubes (Becton Dickinson) containing EDTA by a trained phlebotomist. DNA was extracted using a QIAamp genomic DNA kit (Qiagen, Hilden, Germany) following the manufacturer’s protocol, with the aid of a fully automated QIAcube (Qiagen, Hilden, Germany). The DNA samples were diluted to 5.0ng/μl as per requirements of the SEQUENOM MassARRAY iPLEX® assay protocol.

### Selection and genotyping of SNPs

136 SNPs, located in 66 genes and some undefined chromosomal locations were identified by a thorough literature search of GWAS for both aggressive PCa and PCa. Care was taken to select SNPs that were identified as significantly associated with risks for PCa and aggressive PCa in European populations only. Research papers were only considered if published in and after the year 2000, in order to be in concordance with the current trends in PCa research. The final SNPs selected to be genotyped were at the research team’s discretion, either by SEQUENOM MassARRAY iPLEX ^®^ assay or by TaqMan ^®^ SNP genotyping assay, as discussed in Vaidyanathan *et al.*, 2017 ^18^.

SNP genotyping by SEQUENOM MassARRAY iPLEX ^®^ assay for the candidate SNPs was carried out in the Auckland UniServices Sequenom Facility at The Liggins Institute, Auckland, and AgResearch Limited, Mosgiel, New Zealand, using a custom-designed multiplex gene panel and iPlex chemistry. The genotype calling was carried out by using the standard post-processing calling parameters from the SEQUENOM Type 4.0 software.

SNP genotyping using the TaqMan ^®^ SNP genotyping (Applied Biosystems, ABI) was carried out on a panel of genes that failed to be genotyped using the SEQUENOM MassARRAY iPLEX® assay. The primers used were either obtained pre-designed from ABI or were custom-made using Assay-by-Design service by ABI, and the protocol provided by the manufacturers was followed ^7, 18– 20^.

### Statistical analysis

26 SNPs were removed for being in linkage, and a further 5 SNPs were removed for failing the Hardy-Weinberg Equilibrium (HWE) in the healthy controls, thereby reducing the total SNPs analyzed to 105 (colour coded in Dataset 1 ^17^). SNPs that failed the HWE in patients with PCa were still considered for analysis, as the SNPs may have failed to be in equilibrium in the patient population due to the influence of the risk allele and hence should not be ignored from a case-control study like ours ^21, 22^. The statistical significance was set to p≤0.0001 ^23^.

Analysis of the data for SNP-SNP interactions associated with aggressive PCa was carried out using PLINK software version 1.07 ^18, 23^. PLINK’s clustering approach, identical-by-state (IBS) clustering, is based on pairing up the SNPs based on similarity of genetic identity. This IBS clustering is used in order to test if the SNPs of two individuals belong to the same population or not ^18^. Following this stratification, we performed a standard case-control association test using the Cochran-Mantel-Haenszel test (1 degree of freedom) to analyse the SNP-disease association that is conditional on the clustering ^18^. The slower ‘--epistasis’ command was used to test for epistasis using logistic regression ^23^. It is the most accurate test to define SNP-SNP interactions using PLINK ^23^.

## Results

Table 1 shows the statistically significant SNP-SNP interaction discovered in patients with aggressive PCa when compared to healthy controls. The results obtained for other categorical analyses are not discussed here, as they were not statistically significant in our study and have been mentioned in Supplementary Table 2. The SNP rs2121875, an intronic SNP present in chromosomal position 5p12 near the fibroblast growth factor 10 ( *FGF10*) gene ^24^, has been identified to be associated with the SNP rs4809960, an intronic SNP present in chromosomal position 20q13 near the gene cytochrome P450 family 24 subfamily A member 1 ( *CYP24A1)* ^25^, such that the latter SNP raises the odds of having the prior.

**Table 1.** Statistically significant SNP-SNP interactions discovered in patients with aggressive PCa when compared to healthy controls. CHR1: chromosome of first SNP, SNP1: Identifier for first SNP, CHR2: Chromosome of second SNP, SNP2: Identifier for second SNP, OR_INT: Odds ratio for interaction, STAT: Chi-square statistic 1df, p: Asymptotic p-value.

CHR1	SNP1	CHR2	SNP2	OR_INT	STAT	P
5p12	rs2121875	20q13	rs4809960	2.918	15.77	7.15E-05

Raw data from the current study Click here for additional data file. Copyright: © 2017 Vaidyanathan V et al. 2017 Data associated with the article are available under the terms of the Creative Commons Zero "No rights reserved" data waiver (CC0 1.0 Public domain dedication).

Epistasis results after analysis of the data for SNP-SNP interactions Click here for additional data file. Copyright: © 2017 Vaidyanathan V et al. 2017 Data associated with the article are available under the terms of the Creative Commons Zero "No rights reserved" data waiver (CC0 1.0 Public domain dedication).

## Discussion

Epistatic effects that are crucial to define various biologically-intuitive models of interaction between two SNPs have already been observed in a variety of species ^11^. We believe this is the first study on SNP-SNP interactions associated with aggressive PCa carried out with patients from a New Zealand population.

The SNP rs4809960 in the gene *CYP24A1* has been reported by Holt **et al.**, (2010) to be associated with prostate cancer-specific mortality, and was not evolutionarily conserved ^25^. It was also found to have an effect on the body mass index (BMI), but due to a small sample size the hazard ratios for the BMI strata were not considered reliable enough to be reported ^25^. The protein encoded by *CYP24A1* initiates the degradation of the physiologically active form of Vitamin D3 (VD3) ^26^. VD3 is an important hormone that is actively involved in regulating cell proliferation in the prostate, and has also been identified to have increased expression in PCa cell lines ^27^. It is well established that, with ageing, the skin cannot synthesize VD3 as effectively as desirable and the kidney’s ability to convert VD3 to its active form decreases ^28^. This is of relevance because PCa has always been considered as a disease of elderly men ^29^ who have had less exposure to sunlight and thereby Vitamin D3 ^30^. It is even more intriguing for the other epistatic SNP to be identified in *FGF10*.

According to Paul *et al*. (2013), during mesenchymal development, eFGF10 protein can trigger PCa development through increased androgen receptor expression in the neoplastic epithelium ^31^. It is also worthy to mention that FGF10 is closest to FGF7 based on its evolutionary history ^32^, and according to Emoto **et al.** (1997), is suggested to have no activity for fibroblasts ^32^. We do not agree with this, because fibroblasts in certain organs, senesce due to aging ^33^, and can promote tumour invasion ^34^. This logical progression of ageing-led senescence and promotion of tumour invasion holds true for ageing and risk of aggressive PCa ^16^ as well.

We suggest that the intronic SNP rs2121875 in the gene *FGF10* may be causing alterations in gene expression, perhaps due to the prevalent external/environmental conditions in the elderly men with PCa. Our theory is based on the recent discovery in a study by Zhang *et al*. (2007) that even intronic SNPs (such as the ones identified in *FGF10* and *CYP24A1*) can change the outcome and usage of exons ^35, 36^. This unique and novel epistatic finding emphasizes the fact that intronic SNPs (and SNP-SNP interactions) can also have a significant effect on the risk of diseases such as aggressive PCa, and need to be investigated further.

## Data availability

The data referenced by this article are under copyright with the following copyright statement: Copyright: © 2017 Vaidyanathan V et al.

Data associated with the article are available under the terms of the Creative Commons Zero "No rights reserved" data waiver (CC0 1.0 Public domain dedication). 

Dataset 1: Raw data from the current study. DOI, 10.5256/f1000research.11027.d158605 ^17^

Dataset 2: Epistasis results after analysis of the data for SNP-SNP interactions. DOI, 10.5256/f1000research.11027.d158606 ^37^
