# Supplementary material for: SNP-SNP interactions as risk factors for aggressive prostate cancer
Source: F1000Res. 2017 May 3;6:621. [Version 1] doi: 10.12688/f1000research.11027.1 (PMC5437948; doi:10.12688/f1000research.11027.1)
Supplement: Peer review history

# Peer review: Referee response for version 1

**DOI:** 10.5256/f1000research.11895.r22808

**Published:** 2017-05-17

**Authors:** Yog Raj Ahuja, Zeenath Jehan

**Affiliations:** 1 Department of Molecular Medicine, Vasavi Medical & Research Centre, Hyderabad, Telangana, India

The authors have carried out SNP genotyping in from a New Zealand cohort of men with self-reported European ethnicity that have been clinically diagnosed with aggressive and non-aggressive PCa, and healthy controls. They have identified a number of SNPs from the GWAS from various European populations and described SNP-SNP interaction as a risk factor for aggressive PCa in a New Zealand cohort. The intronic SNP rs2121875, an on chromosomal position 5p12 near the fibroblast growth factor 10 ( *FGF10*) gene24, has been identified to be associated with the intronic SNP rs4809960, an intronic SNP present in chromosomal position 20q13 near the gene cytochrome P450 family 24 subfamily A member 1 ( *CYP24A1*). The protein encoded by *CYP24A1* initiates the degradation of the physiologically active form of Vitamin D3 (VD3) 26 which is an important hormone that is actively involved in regulating cell proliferation in the prostate, and has also been identified to have increased expression in PCa cell lines. The epistatic effect of the SNP-SNP interactions suggested by the authors may be relevant in view of many recent studies showing intronic mutations which can exert their effect on protein coding exons. The recent observation of decreasing Vitamin D3 levels worldwide further support the role of environmental factors in these gene environment interactions. Future studies may help in understanding the role of SNPs and environmental interactions.

We have read this submission. We believe that we have an appropriate level of expertise to confirm that it is of an acceptable scientific standard.

# Peer review: Referee response for version 1

**DOI:** 10.5256/f1000research.11895.r22484

**Published:** 2017-05-09

**Authors:** Syed Muhammad Shahid

**Affiliations:** 1 The Karachi Institute of Biotechnology and Genetic Engineering (KIBGE), University of Karachi, Karachi, Pakistan

The manuscript identified a number of SNPs in GWAS as risk factors for PCa in various European populations as well as defined SNP-SNP interactions, using PLINK software, with nucleic acid samples from a New Zealand cohort. The approach which authored used to find a gene x environment marker for aggressive PCa gene x environment interactions can be adjusted statistically, however, it is highly impossible in practicality.

 The manuscript compiled most of recent literature available on the subject and propose justified discussion on the research question. 

 Since I do not have sufficient expertise in statistical analyses used to elaborate the key findings of the manuscript, I am reluctant to comment on the authenticity and validity of conclusion.

I have read this submission. I believe that I have an appropriate level of expertise to confirm that it is of an acceptable scientific standard.
